# The application of tranexamic acid in joint arthroplasty: A 20-year bibliometric analysis

**DOI:** 10.3389/fpubh.2022.1013461

**Published:** 2022-11-01

**Authors:** Jun Zhang, Runhan Zhao, Yanran Huang, Chuang Xiong, Hao Liang, Habu Jiwa, Xiaoji Luo

**Affiliations:** ^1^Department of Orthopedics, The First Affiliated Hospital of Chongqing Medical University, Chongqing, China; ^2^Orthopedic Laboratory of Chongqing Medical University, Chongqing, China

**Keywords:** tranexamic acid, joint arthroplasty, bibliometric approach, trends, hotspots

## Abstract

**Background:**

With the arrival of the era of the aging population, the amount of joint arthroplasty surgery keeps rising, and the articles related to the application of tranexamic acid (TXA) in joint arthroplasty (we called the application of tranexamic acid in joint arthroplasty as TIA in this study) also show a blowout growth. Therefore, we conducted a bibliometric analysis of TIA-related publications to identify the main research trends and hot spots in this field in the last 20 years.

**Methods:**

In this study, publications in the field of TIA from January 1, 2002 to December 31, 2021 were searched in the Web of Science Core Collection (WoSCC). A total of 1,013 publications were evaluated for specific characteristics with Microsoft Excel software, CiteSpace, VOSviewer, and Online Analysis Platform of Literature Metrology (http://bibliometric.com/).

**Results:**

A total of 1,013 TIA-related articles were included in this study, and the number of articles in this field has increased yearly over the past 20 years. The USA and China dominated in the field of TIA. The Sichuan University published the most TIA-related articles among all the institutions. Of all the authors, Professor Pei was the most productive author with 64 articles. The lack of international cooperation was a significant problem in this field during the past 20 years. Furthermore, the results of the co-citation analysis and citation bursts analysis revealed that the safety and effectiveness of TIA and the optimal use strategy were the main trends and hotspots for the current and future.

**Conclusion:**

This bibliometric study reviewed the evolution trend of TIA research, and identified the countries, institutions, authors and journals that have made significant contributions to this field in the past 20 years, as well as the limitations and deficiencies in this field. In addition, this study revealed that the effectiveness and safety of TIA and the optimal use strategy was the current or future research trend and hotspot in this field.

## Introduction

Joint arthroplasty is considered one of the most successful operations in all medicine, which can not only relieve the patient's symptoms but also improve the quality of life of patients (1). And the two most common types of joint arthroplasty are hip arthroplasty and knee arthroplasty, which significantly improve the life quality of patients with osteoarthritis or other joint diseases (2). Reducing the perioperative blood loss and transfusion demand of the joint arthroplasty has always been the main problem clinicians always face. Over the past few decades, many interventions have been developed to address this problem, such as controlled hypotensive anesthesia (3), blood recovery techniques (4), drain clamping (5), as well as the application of the erythropoietin and antifibrinolytic agents (6, 7). Tranexamic acid (TXA) is an antifibrinolytic drug that stops bleeding by activating plasminogen to induce thrombosis (8, 9). It has been successfully utilized to reduce bleeding in cardiac surgery (10), liver surgery (11), and gynecology (12). Therefore, the application of TXA in joint arthroplasty (TIA) has also been under the close attention of researchers. In recent years, with the increase in the volume of joint arthroplasty operations (13, 14), the TIA-related articles showed explosive growth.

At present, there are many studies on the perioperative application of TXA in joint arthroplasty, but there is no bibliometric analysis to summarize the literature on this field. As a unique statistical method, bibliometric analysis is mainly utilized to analyze and visualize critical features of published articles in a field (15). Based on the bibliometric analysis, we can accurately identify the most influential authors, journals, articles, institutions, or countries/regions in this field and determine the research hotspots and trends in this field. Currently, bibliometric analysis has been widely used in a variety of fields, including health care, environmental science, energy management, and so on (16–24). However, no study has summarized and analyzed the trends in the field of TIA by using bibliometric analysis. Therefore, this study conducted a comprehensive bibliometric analysis of the articles on TIA-related research from 2002 to 2021, which aimed to deepen the understanding of the research hotspots and potential trends in this field and provide helpful reference guidelines for future researchers.

## Materials and methods

### Sources of data and search strategy

Because the Web of Science Core Collection (WoSCC) of Clarivate Analytics is the most frequently used scientific information source, the literature of this bibliometric analysis study all obtained from the Science Citation Index Expanded (SCIE) and the Social Science Citation Index (SSCI) of the WoSCC. Moreover, the search strategy was as follows: TS = (“Tranexamic Acid” OR Transamin OR Cyklokapron) AND TS = (Arthroplasty OR “Total Knee Arthroplasty” OR “Total Knee Replacement” OR “Knee Arthroplasty” OR “Arthroplasty Knee” OR “Replacement Total Knee” OR “Knee Replacement Total” OR “Unicompartmental Knee Arthroplasty” OR “Arthroplasty Unicompartmental Knee” OR “Knee Arthroplasty Unicompartmental” OR “Unicondylar Knee Arthroplasty” OR “Arthroplasty Unicondylar Knee” OR “Knee Arthroplasty Unicondylar” OR “Partial Knee Arthroplasty” OR “Arthroplasty Partial Knee” OR “Knee Arthroplasty Partial” OR “Unicondylar Knee Replacement” OR “Knee Replacement Unicondylar” OR “Partial Knee Replacement” OR “Knee Replacement Partial” OR “Unicompartmental Knee Replacement” OR “Knee Replacement Unicompartmental” OR“Arthroplasties Knee Replacement” OR “Replacement Arthroplasty Knee” OR “Arthroplasty Replacement Partial Knee” OR “Arthroplasties Replacement Knee” OR “Arthroplasty Knee Replacement” OR “Knee Replacement Arthroplasties” OR “Knee Replacement Arthroplasty” OR “Replacement Arthroplasties Knee” OR “Knee Arthroplasty Total” OR “Arthroplasty Total Knee” OR “Arthroplasty Hip Replacement” OR “Hip Replacement Arthroplasty” OR “Replacement Arthroplasties Hip” OR “Replacement Arthroplasty Hip” OR “Total Hip Replacement” OR “Total Hip Arthroplasty” OR “Arthroplasty Total Hip” OR “Hip Arthroplasty Total” OR “Total Hip Arthroplasties” OR “Replacement Total Hip” OR “Total Hip Replacements” OR “Arthroplasties Hip Replacement” OR “Hip Replacement Arthroplasties” OR “Hip Replacement Total” OR “Arthroplasties Replacement Hip” OR “Hip Prosthesis Implantation” OR “Hip Prosthesis Implantations” OR “Implantation Hip Prosthesis” OR “Prosthesis Implantation Hip”). The document type was set as “Articles,” the publication time was limited to a period from 2002 to 2021, and the language was set as “English.”

### Article screening

**Figure 1:**
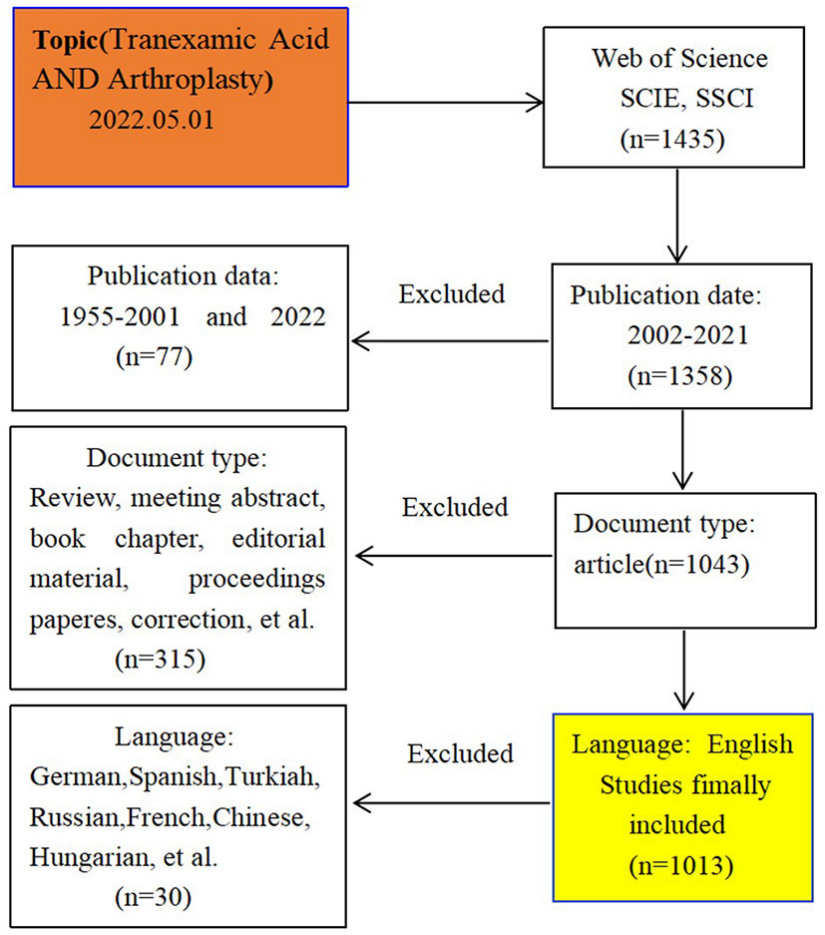
Flowchart of study identification and selection based on Web of Science.

The data were collected on May 1, 2022, and 1435 original articles were obtained from the WoSCC. After screening, 1,013 articles were included in this analysis, while 412 articles were excluded because they did not meet the requirements: publication time exceeded the set range (*n* = 77), the document type was not “Article” (*n* = 315), and the language is not “English” (*n* = 30). The research characteristics of these publications were shown in Figure 1. In this study, two researchers independently performed the data research and resolved disagreements by mutual agreement.

### Bibliometric analysis

The 1,013 articles were marked on the Web of Science website. The following information for each article was extracted: authors, journals, institutions, countries, keywords, and so on. Then, these data were imported into the CiteSpace, the VOSviewer, Microsoft Excel, and the Online Analysis Platform of Literature Metrology (http://bibliometric.com/) to perform data processing and network visualization analysis. The VOSviewer and the CiteSpace software were the two main softwares used in bibliometric analysis in this study. Among them, the VOSviewer is the most frequently used, and it can explore collaborative networks between authors/institutes/countries/journals and visualize the results (25). The CiteSpace is a visualization software developed by Chen to extract keywords and references from publications with high citation bursts and construct a dual-map overlay for journals (26).

## Results

### Global publication outputs and citations

**Figure 2:**
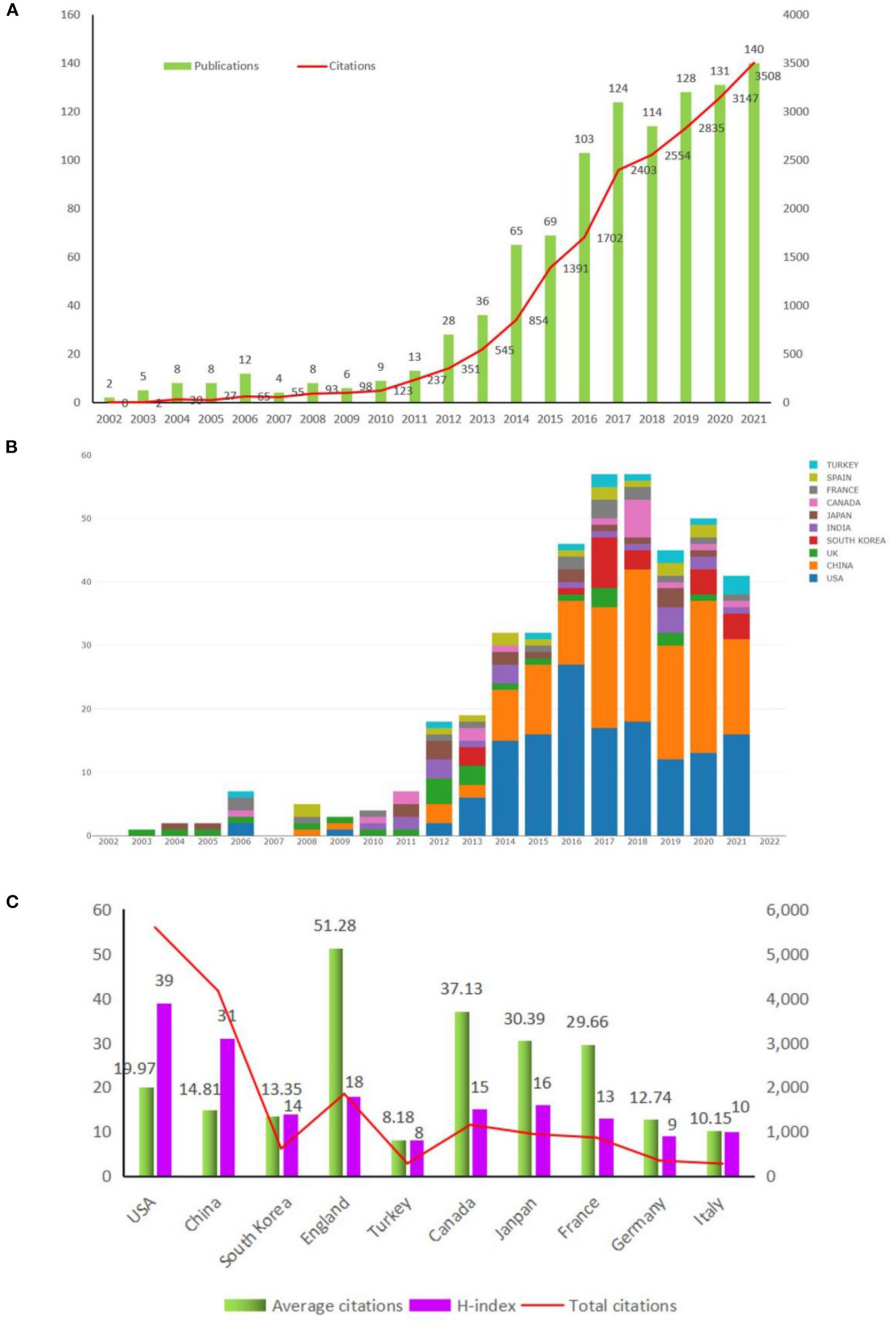
The number of publications, the number of citations, and the H-index for TIA-related research. **(A)** The annual cumulative number of publications; **(B)** The annual number of publications in major countries; **(C)** The annual citation number and the annual H-index of publications.

From 2002 to 2021, we observed a clear upward trend in the number of publications and citations, which indicated that the TIA field has attracted more and more attention from researchers (Figures 2A,B). In the past two decades, TIA-related articles were mainly from East Asia, Europe, and America. And the characteristics of the top 10 active countries are shown in Table 1.

**Table 1:** The top 10 countries that contributed publications on the TIA field.

**Rank**	**Country**	**Total publications**	**Total** **citations**	**Average citations**	**H-index**
1	China	281	4,163	14.81	31
2	USA	280	5,592	19.97	39
3	South Korea	46	614	13.35	14
4	England	36	1,846	51.28	18
5	Turkey	34	278	8.18	8
6	Canada	31	1,151	37.13	15
7	Japan	31	942	30.39	16
8	France	29	860	29.66	13
9	Germany	27	344	12.74	9
10	Italy	27	274	10.15	10

**Figure 3:**
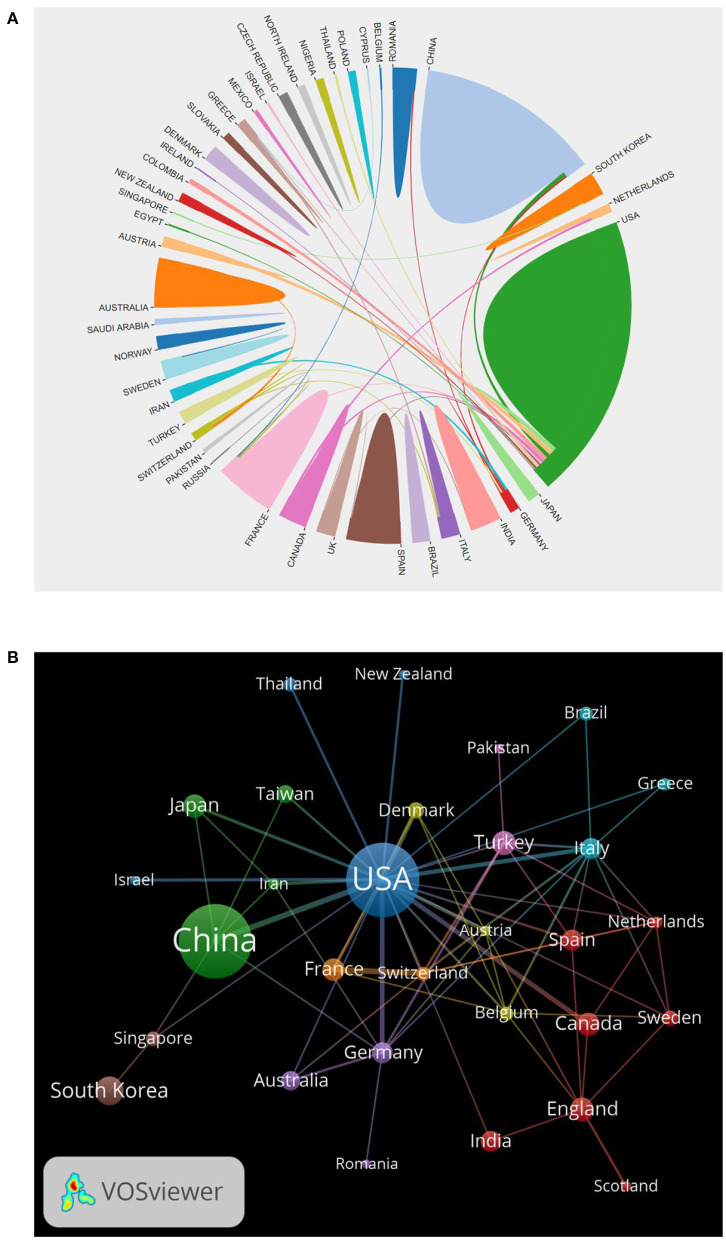
Country analysis. **(A)** Co-operation between countries/regions; **(B)** Network map of co-authorship country.

Of these, China and USA were the most productive country, of which China published 281 articles and the USA published 280 articles. Figure 2C showed that the two countries both owned high citations and H-index values, which proved their significant scientific influence of them in this field. Subsequent network correlation analysis also confirmed that the USA and China were the most active countries in TIA-related research, and there was a relatively high collaboration between Germany and the USA and between China and the USA (Figures 3A,B). However, researches among other countries were relatively independent, and international cooperation needed to be strengthened (Figure 3B).

### Institutions analysis

**Figure 4:**
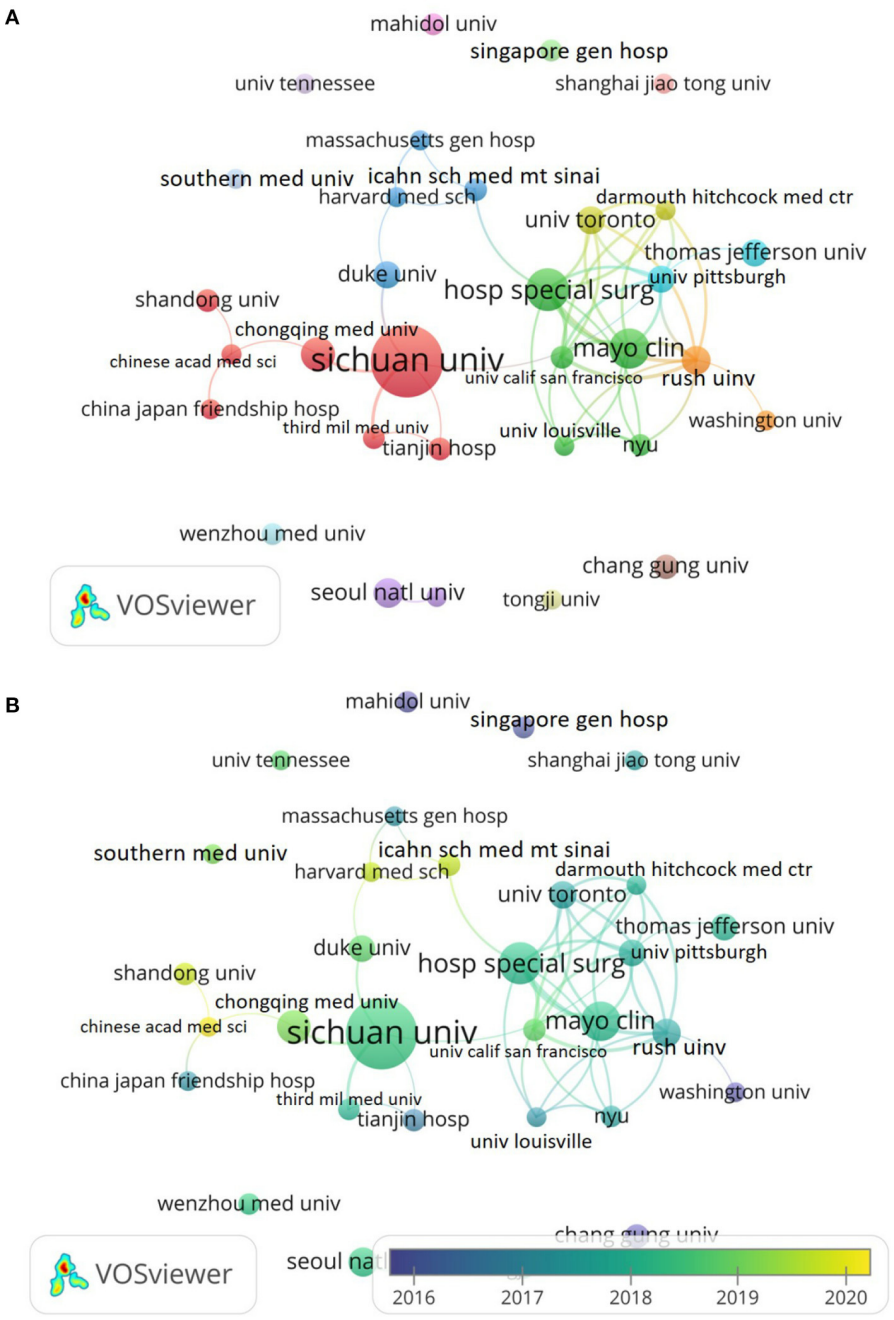
Institution analysis. **(A)** Network map of interinstitutional cooperation; **(B)** Dynamic graph of temporal trends of articles published in the TIA field.

Table 2 showed the top 10 institutions in terms of publication number, total citations, average citations and H-index. Sichuan University topped the list with a total of 85 publications and 1,490 citations, followed by the Hospital for Special Surgery (31 publications), the Mayo Clinic (28 publications), the Chongqing Medical University (19 publications), and the Rush University (14 publications). The cooperation network showed that the Sichuan University owned a much greater influence on TIA-related research than any other institutions, and the cooperation network also showed that the cooperation among institutions was mostly limited to their respective countries while international cooperation was rare (Figure 4A). The network analysis also showed that in recent years, articles in the TIA field mainly came from China, the US and the UK, and that Sichuan University remained active in this field (Figure 4B).

**Table 2:** The top 10 institutions that contributed publications on the TIA field.

**Rank**	**Institution**	**Country**	**Total publications**	**Total** **citations**	**Average** **citations**	**H-index**
1	Sichuan University	China	85	1,490	17.53	20
2	Hospital For Special Surgery	USA	31	827	26.68	14
3	Mayo Clinic	USA	28	962	34.36	18
4	Chongqing Medical University	China	19	247	13	8
5	Rush University	USA	14	504	36	10
6	Jefferson University	USA	16	210	13.13	9
7	Seoul National University Snu	South Korea	15	196	13.07	8
8	University of Toronto	USA	16	757	47.31	9
9	University of Pittsburgh	USA	12	518	43.17	9
10	Duke University	USA	12	165	13.75	8

### Contributions of authors

**Figure 5:**
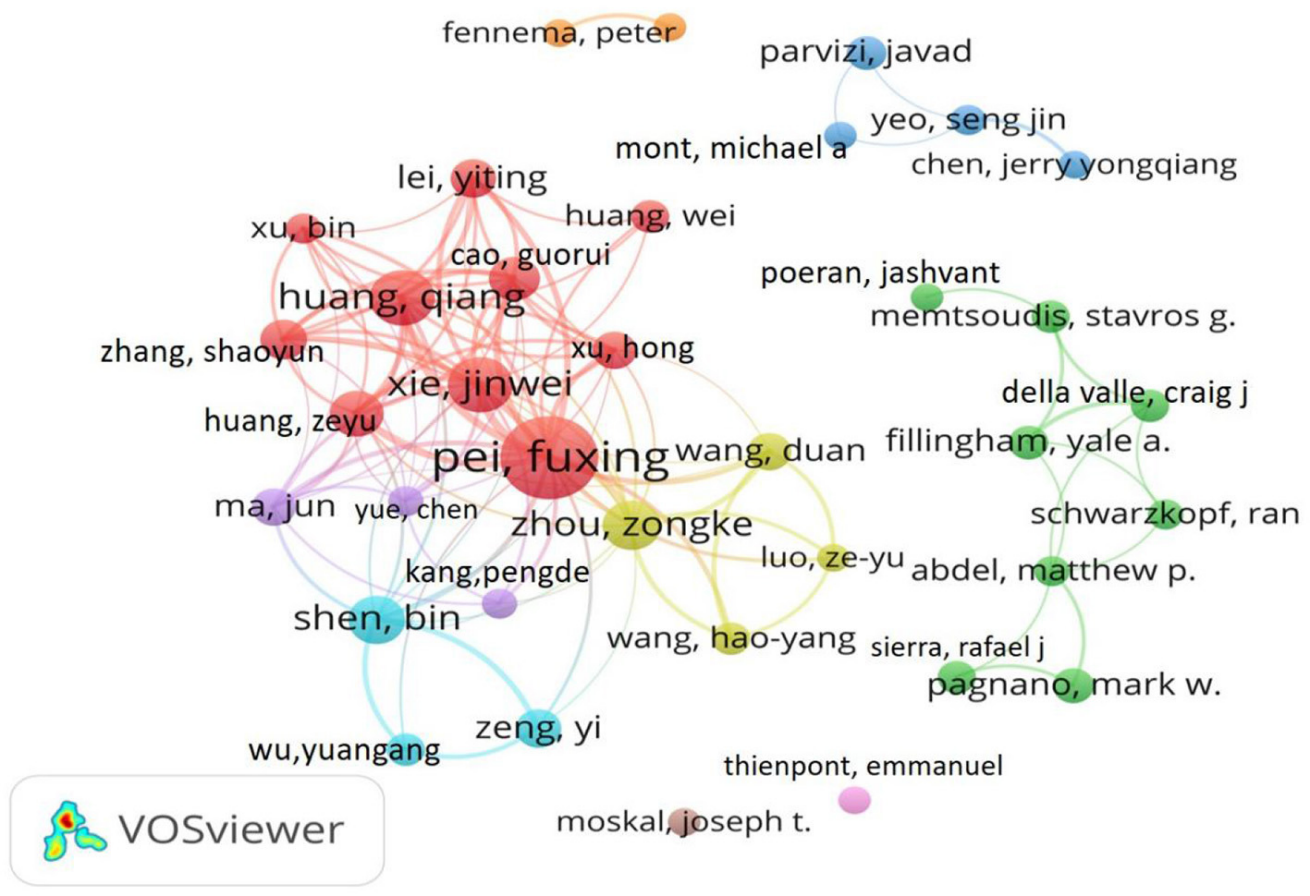
Network map of author collaboration.

Based on the H-index and the number of publications, we identified the 10 most influential authors of TIA research (Table 3). The top five most productive authors were Pei Fuxing (published 64 articles), followed by Huang Qiang (published 28 articles), Xie Jinwei (published 27 articles), Zhou Zongke (published 26 articles), and Shen Bin (published 21 articles). Among them, Pei Fuxing's total number of publications, the total number of citations, average number of citations, and H-index all remained at the top 1, indicating that he had a significant influence in this field. In addition, we also analyzed the collaborative relationships among highly productive authors in the field (Figure 5). Based on this collaborative map, we identified several key research teams (Pei Fuxing; Xie Jinwei; and Huang Qiang) and found that all of them had close cooperation.

**Table 3:** The top 10 authors that contributed publications on TIA field.

**Rank**	**Authors**	**Total publications**	**Total citations**	**Average citations**	**H-index**
1	Pei Fuxing	64	1,285	20.08	20
2	Huang Qiang	28	497	17.75	14
3	Xie Jinwei	27	623	23.07	13
4	Zhou Zongke	26	452	17.38	12
5	Shen Bin	21	498	23.71	12
6	Huang Zeyu	20	371	18.55	12
7	Cao, guorui	16	189	11.81	9
8	Xu Hong	16	118	7.38	7
9	Lei Yiting	15	236	15.73	9
10	Wang Duan	15	238	15.87	11

### Contributions of journals

**Figure 6:**
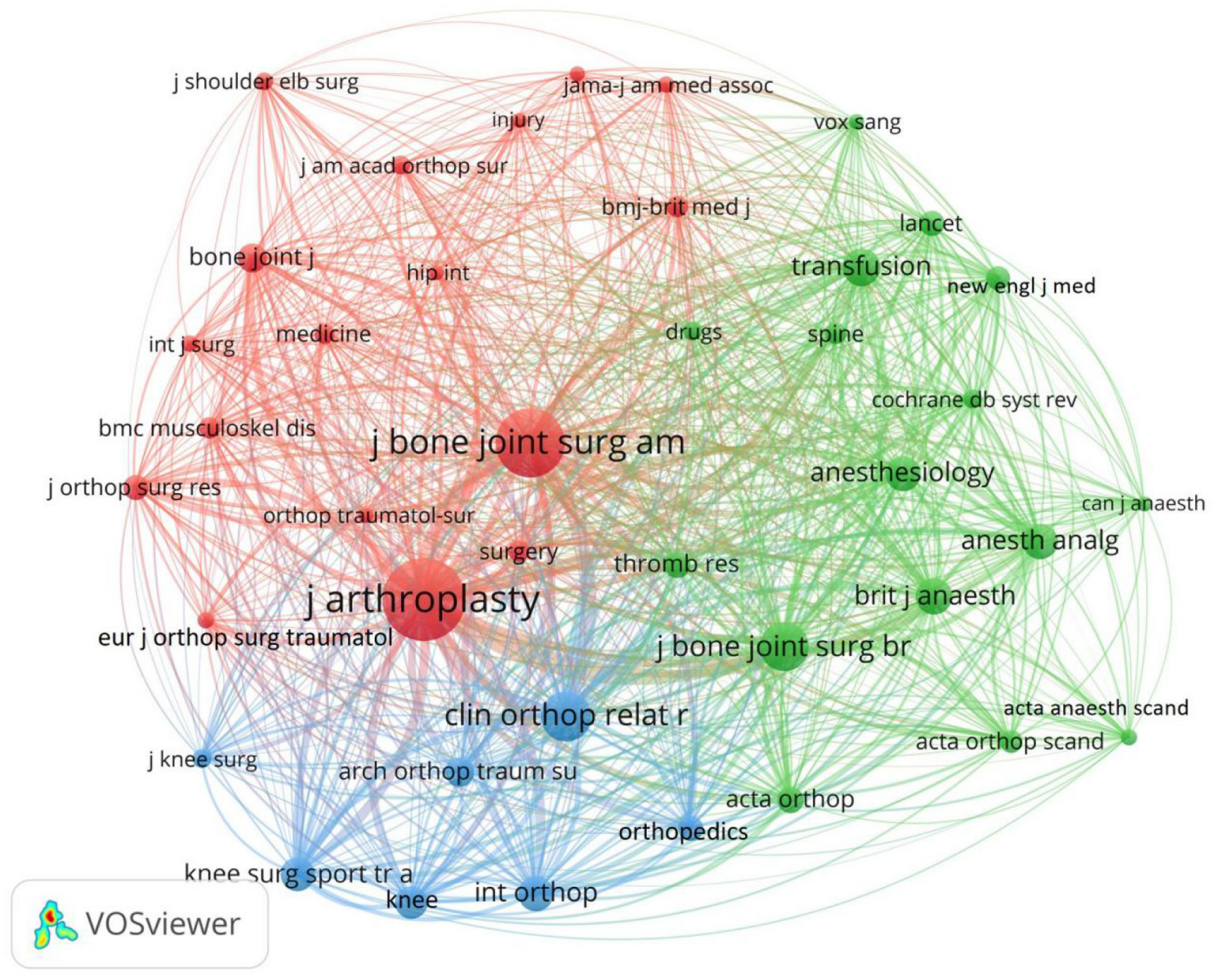
Visualization map of co-cited journals.

Table 4 listed the 10 most active journals and the 10 most influential journals in the field of TIA research. Most of the top 10 active journals belonged to the USA, followed by the UK and Germany. The top 3 active publications in the past 20 years were *Journal of Arthroplasty, BMC Musculoskeletal Disorders*, and *International Orthopedics*. Moreover, in these journals, *Journal of Arthroplasty, Journal of Bone and Joint Surgery American Volume*, and *Knee Surgery Sports Traumatology Arthroscopy* were ranked as Q1 in the Journal Citation Report quartile; *BMC Musculoskeletal Disorders, International Orthopedics, Journal of Orthopedic Surgery and Research, Archives of Orthopedic and Trauma Surgery, Bone Joint Journal*, and *Medicine* were ranked in Q2. *Journal of Knee Surgery* was ranked in Q3. This result proved that articles in this field had high quality and were generally recognized by peers. Furthermore, the citations of *Journal of Arthroplasty* and *Journal of Bone and Joint Surgery American Volume* were relatively high, indicating that the manuscripts published in these two journals were of high quality and well-recognized by peers. Subsequent network analysis further confirmed these two journals' strong influence on TIA research (Figure 6).

**Table 4:** Top 10 active journals and top 10 influential journals in the field of TIA.

**Journal**	**Articles**	**Country**	**JCR**	**IF**	**Co-cited journal**	**Co-citations**	**JCR**	**IF**
Journal of Arthroplasty	160	USA	Q1	4.757	Journal of Arthroplasty	3,971	Q1	4.757
BMC Musculoskeletal Disorders	41	England	Q2	2.362	Journal of Bone and Joint Surgery American Volume	2,745	Q1	5.284
International Orthopedics	36	USA	Q2	3.075	Clinical Orthopedics and Related Research	1,471	Q1	4.176
Journal of Orthopedic Surgery And Research	34	England	Q2	2.359	Journal of Bone and Joint Surgery British Volume	1,436	–	–
Journal of Bone and Joint Surgery American Volume	33	USA	Q1	5.284	Transfusion	870	Q3	3.157
Journal of Knee Surgery	32	USA	Q3	2.757	British Journal of Anesthesia	793	Q1	9.166
Knee Surgery Sports Traumatology Arthroscopy	27	Germany	Q1	4.342	Anesthesia and Analgesia	749	Q2	5.108
Archives of Orthopedic And Trauma Surgery	26	Germany	Q2	3.067	Anesthesiology	736	Q1	7.892
Bone Joint Journal	24	England	Q2	5.082	International Orthopedics	732	Q2	3.075
Medicine	23	USA	Q2	1.889	Knee Surgery Sports Traumatology Arthroscopy	701	Q1	4.342

**Figure 7:**
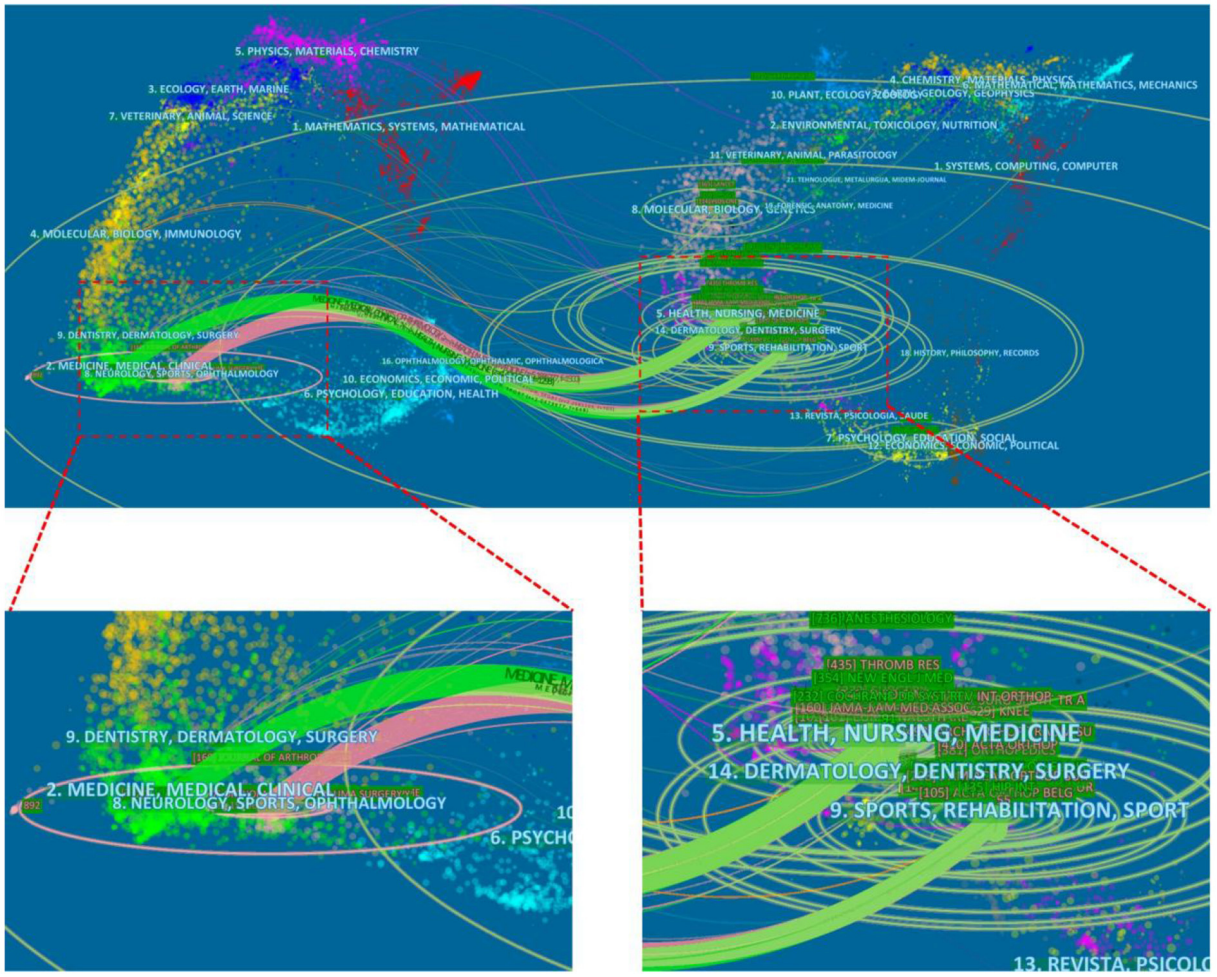
The dual-map overlay of journals related to TIA field based on CiteSpace.

The dual map overlay of journals showed two main citation paths: green and pink (Figure 7). The two paths indicated that papers published in Medicine/Medical/Clinical or Neurology/Sports/Ophthalmology journals usually cited papers published in Healthy/Nursing/Medicine and Sports/Rehabilitation/Sport. This result provided a certain reference for new researchers who began to researches in this field.

### Co-cited articles and co-cited reference cluster analysis

**Figure 8:**
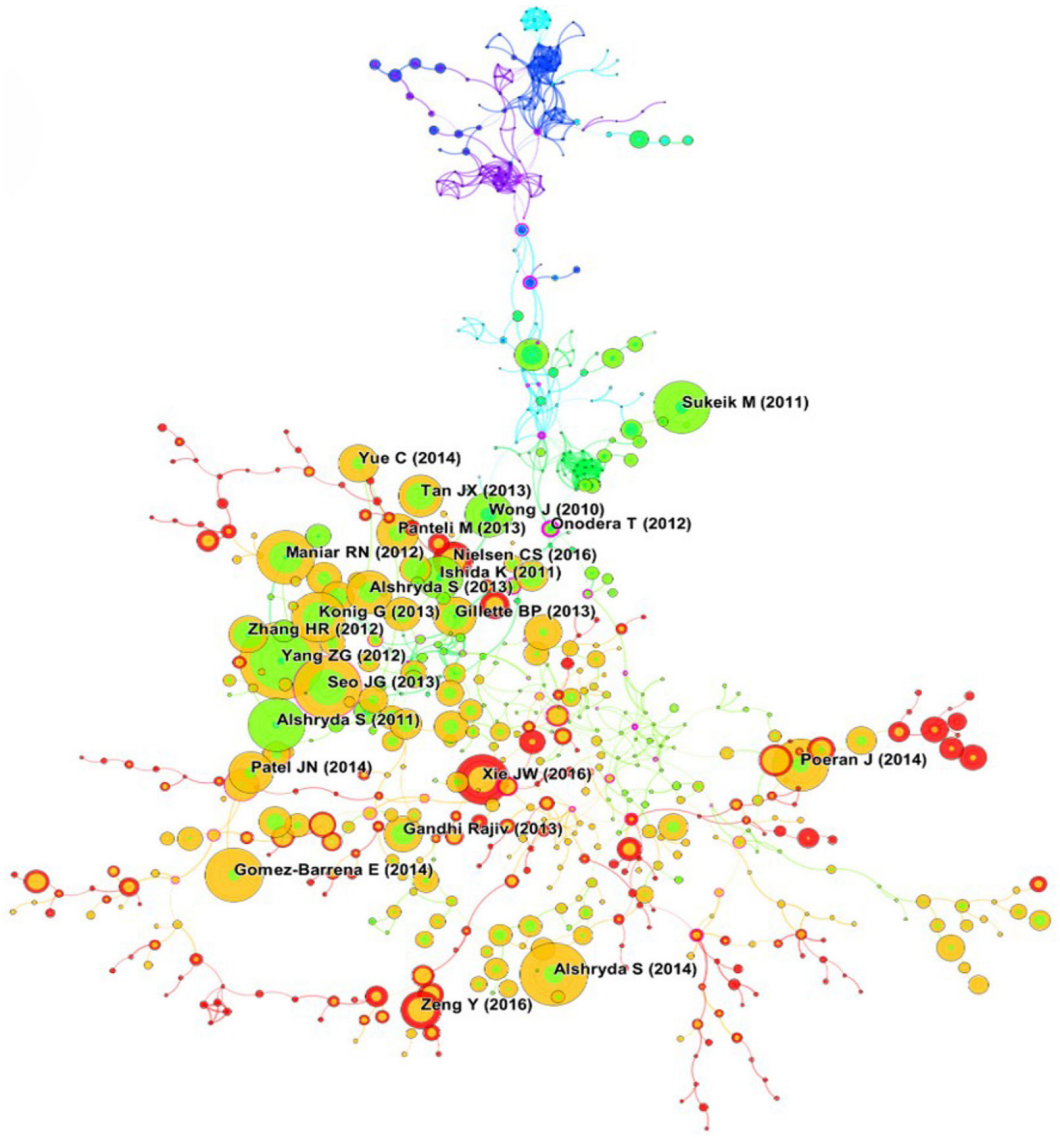
Visualization map of co-cited references.

**Figure 9:**
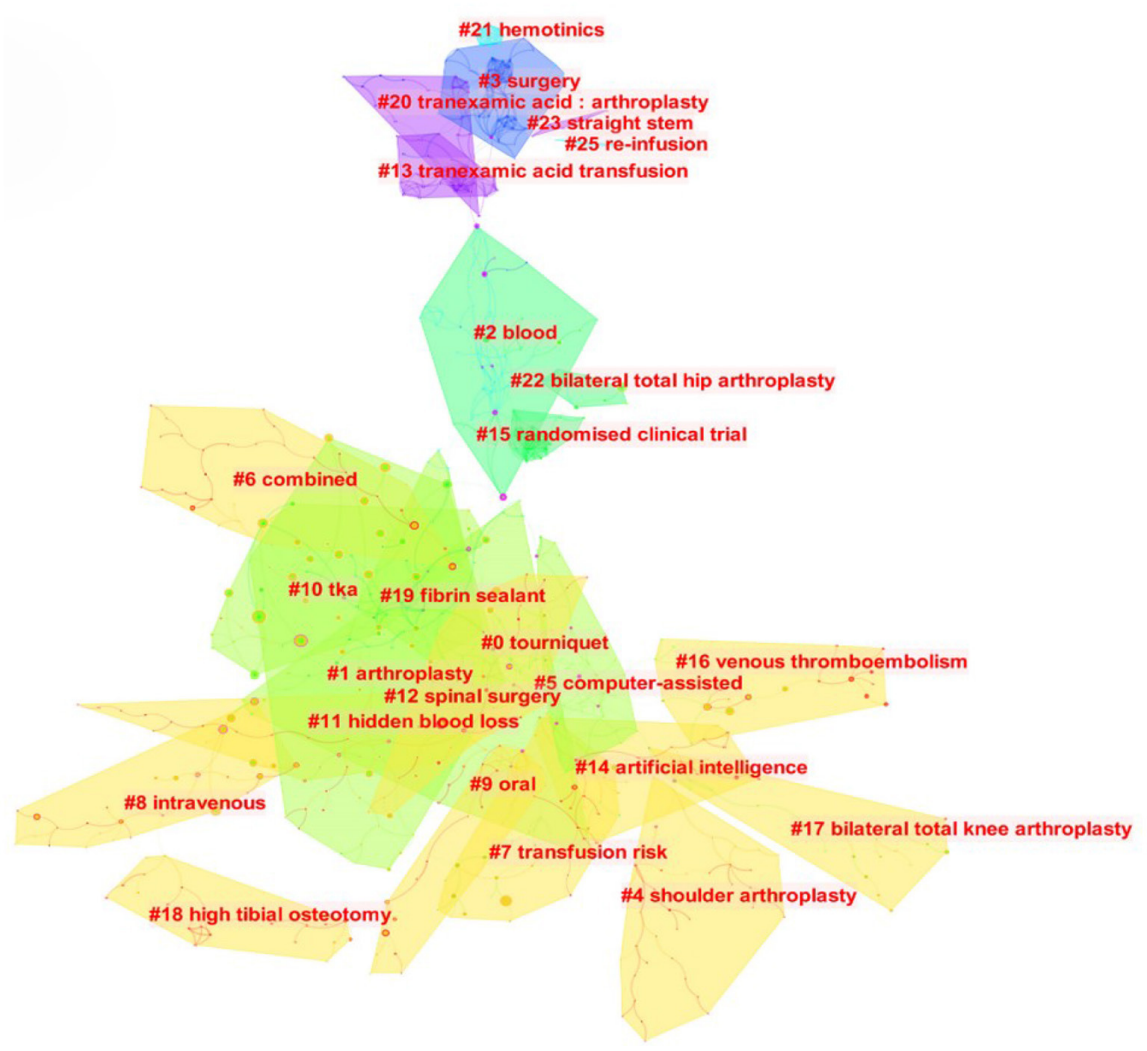
Visualization map of co-cited reference cluster.

Literature co-citation analysis is vital to trace the scientific frontiers and research basis. Co-citation relationship indicates that two or more articles are cited by one or more articles at the same time, which has been widely used as a research method to assess the degree of relationship between different articles. Therefore, we constructed co-citation correlation and cluster network map by using Citespace software. A total of 12,500 cited references were retrieved and the most frequently cited references were shown in Figure 8. For the cluster map (Q = 0.8675, S = 0.9632), we found that the ten hot spots on TIA research in the recent 20 years included: #0 “Tourniquet,” #1 “Arthorplastyt,” #2 “Blood,” #3 “Surgery,” #4 “Shoulder arthroplasty,” #5 “Computer-assisted,” #6 “Combined,” #7 “Transfusion risk,” #8 “Intravenous,” #9 “Oral” (Figure 9). In general, Q > 0.3 and S > 0.5 indicates that the cluster quality of the cluster map was good. Hence the result of the cluster map in this study was convincing. Table 5 listed the top 10 most cited articles, most of which were published in the *J Bone Joint Surg Br, J Bone Joint Surg Am and J Arthroplasty*. Of these, the article entitled *Effectiveness and safety of tranexamic acid in reducing blood loss in total knee arthroplasty: a meta-analysis* was the most frequently cited (*n* = 102) and was published in 2012 by Yang et al. (27). The remaining nine articles were published in the period of 2011–2016, with the number of citations ranging from 67 to 84. Of these 10 studies, six explored the efficacy and safety of TIA. Among them, a retrospective study by Poeran et al. demonstrated the efficacy and the safety of intravenous TXA (28). And the safety and efficacy of topical application of TXA were confirmed in a prospective trial by Konig et al. (29). In addition, the results of Meta analyses by Yang et al. (27), Alshryda et al. (30, 31), and Sukeik et al. (8) all demonstrated an excellent clinical benefit for both intravenous and topical use of TIA, and neither resulted in an increase in associated complications. The remaining four studies were all prospective and focused on exploring the best strategies for the use of TXA. Here, the results of Maniar's et al. (32) and Xie's et al. (33) studies demonstrated a significant reduction in transfusion requirements and blood loss and a better clinical benefit with multiple doses of TXA. The study of Seo et al. showed that topical TXA was superior to intravenous TXA, but Gomez-Barrena et al. demonstrated no significant difference in the clinical benefit of topical application TXA or intravenous application TXA (34, 35). In conclusion, the safety and efficacy of topical or systemic TXA have been confirmed by most studies, but more research is needed to explore its optimal dose and mode of administration.

**Table 5:** Top 10 co-cited references in the fields of TIA.

**Author**	**Citations**	**Title**	**Journal**	**Year**
Zhi-Gao Yang	102	Effectiveness and safety of tranexamic acid in reducing blood loss in total knee arthroplasty: a meta-analysis	J Bone Joint Surg Am	2012
Jai-Gon Seo	84	The comparative efficacies of intra-articular and IV tranexamic acid for reducing blood loss during total knee arthroplasty	Knee Surg Sports Traumatol Arthrosc	2013
Alshryda S	83	A systematic review and meta-analysis of the topical administration of tranexamic acid in total hip and knee replacement	Bone Joint J	2014
Maniar Rajesh N	72	Most effective regimen of tranexamic acid in knee arthroplasty: a prospective randomized controlled study in 240 patients	Clin Orthop Relat Res	2012
Enrique Gomez- Barrena	71	Topical intra-articular compared with intravenous tranexamic acid to reduce blood loss in primary total knee replacement: a double-blind, randomized, controlled, non-inferiority clinical trial	J Bone Joint Surg Am	2014
Alshryda S	70	Tranexamic acid in total knee replacement: a systematic review and meta-analysis	J Bone Joint Surg Br	2011
Poeran Jashvant	70	Tranexamic acid use and postoperative outcomes in patients undergoing total hip or knee arthroplasty in the United States: retrospective analysis of effectiveness and safety	BMJ	2014
Sukeik M	69	Systematic review and meta-analysis of the use of tranexamic acid in total hip replacement	J Bone Joint Surg Br	2011
Jin-Wei Xie	68	Multiple Boluses of Intravenous Tranexamic Acid to Reduce Hidden Blood Loss After Primary Total Knee Arthroplasty Without Tourniquet: A Randomized Clinical Trial	J Arthroplasty	2016
Gerhardt Konig	67	Topical tranexamic acid reduces blood loss and transfusion rates in total hip and total knee arthroplasty	J Arthroplasty	2013

### Emerging trends

**Figure 10:**
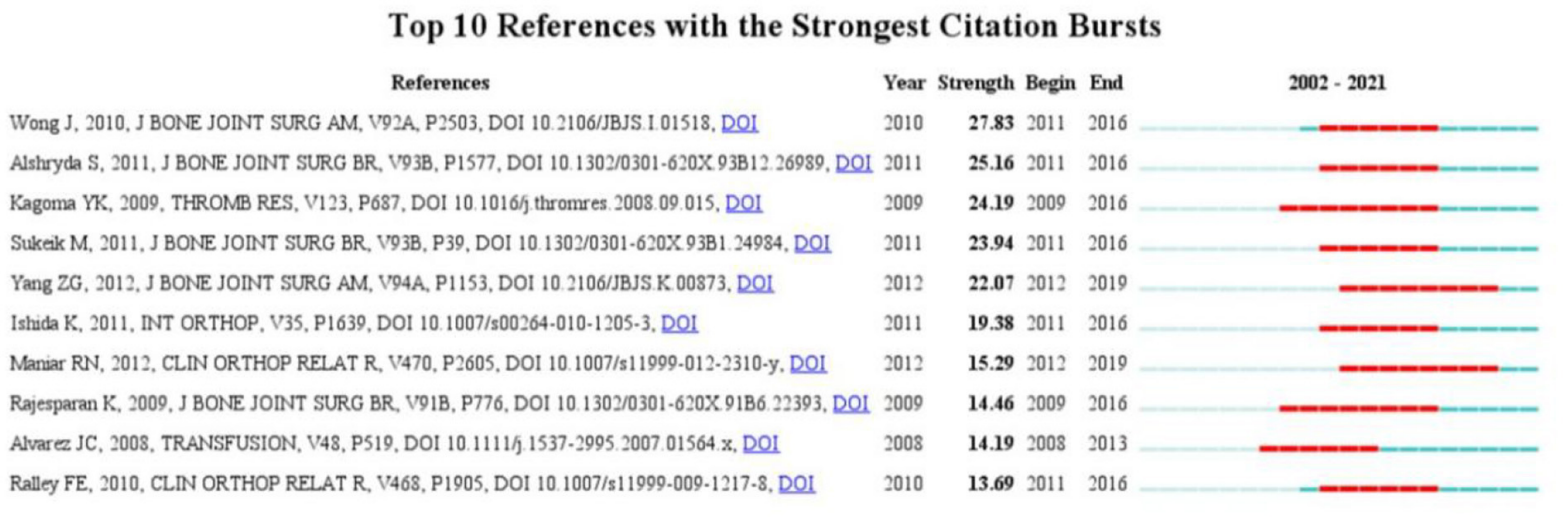
Top 10 references with the strongest citation bursts.

An article with a high citation burst indicated that it had received significant attention from scientific peers and represented an emerging trend or topic in the research field. Herein, we determined the top 10 references with the strongest strength citation bursts in the past 20 years based on the Citespace software. The analysis showed that the burst strength of these papers ranged from 13.69 to 27.83, and the burst duration ranged from 6 to 8 years (Figure 10). Of these papers, the first-ranked paper was published in 2010 and had a burst strength of 27.83 with 6 years of burst duration. This study demonstrated that the local application of TXA to surgical wounds could effectively reduce postoperative bleeding after TKA (36). The burst of the second-ranked paper was maintained for 6 years (2011–2016), and this study demonstrated that intravenous TXA significantly reduced perioperative blood loss and transfusion after TKA without significantly increasing harm (31). As for the third-ranked article, its burst strength was 24.19 and lasted from 2009 to 2016. The article was a systematic review. By integrating articles related to TIA, this study revealed that TXA could significantly reduce the amount of bleeding and the number of transfusions after joint arthroplasty (37). In addition, two papers whose citation burst both ended in 2019 also deserved further discussion. The first article was published in 2012, and the citation burst lasted for 8 years (2012–2019). And it was also the most cited article in the last 20 years. This study confirmed the safety and efficacy of TIA through meta-analysis (27). The second article also lasted for 8 years (2012–2019). This study confirmed that multi-stage perioperative use of TXA could effectively reduce postoperative drainage and total blood loss in TKA patients (32). In conclusion, exploring the security and effectiveness of TXA and the best use strategies are the main hot spots and trends in the TIA field at the moment and even for some time to come.

## Discussion

Perioperative blood loss of joint arthroplasty has always been one of the most concerning problems for orthopedic surgeons. Previous studies showed that the average perioperative blood loss of joint arthroplasty was about 1,450–1,790 ml, and 10–38% of patients needed allogeneic transfusion therapy (38–40). However, the blood transfusion may not only cause multiple serious complications, including infection, immune response, disease transmission, cardiovascular dysfunction, and even death, but also increase treatment costs (6, 30, 41, 42). In 2016 alone, total expenditures related to joint arthroplasty in the United States were ~38 billion dollars (43). As society gradually aging, this number will be increasing year by year. In order to reduce the perioperative transfusion rate of joint arthroplasty, researchers have developed numerous interventions, including controlled hypotensive anesthesia, various blood recovery techniques, and antifibrinolytic drugs (3, 6, 7). Among them, the application of TXA has achieved excellent clinical efficacy and cost-effectiveness, which is favored by researchers (44–46). We noticed an explosion in the number of TIA-related publications since 2011, which showed that the field was getting more attention. Therefore, we conducted a bibliometric analysis with the aim of conducting a preliminary exploration of the field as well as tracking research trends and the latest hot spots in TIA-related field.

A total of 1,013 TIA-related articles were included in this study. We found that, since 2011, the number of TIA-related publications has increased rapidly. Among them, China and the USA accounted for the vast majority. Moreover, China and the USA also ranked top in the total citation and H-index, indicating that these two countries occupied a leading position in the TIA field. In addition, the UK ranked first in the average citations, indicating that the country's research was of high quality. As for international cooperation, the research of countries in the TIA field was relatively independent, and only Germany, the USA and China had relatively high cooperation frequency. Therefore, international cooperation need to be strengthened in the field of TIA.

Through the analysis of institutions and authors, we could understand the contribution of researchers/institutions to this field and their influence on the field of TIA. The results indicated that Sichuan University played a crucial role in the TIA field. Compared to other institutions, the number, total citations, and H-index of TIA-related articles published by Sichuan University ranked first. The eighth institution was the University of Toronto, which published 16 articles in the field of TIA. What's more, the TIA-related articles published by this institute ranked first in the average citations, indicating that this institution's research was of high quality. In terms of author analysis, scholars from China dominated the TIA field. Professor Pei Fuxing had the strongest influence in this field, who ranked first in the total publications, citations, and H-index. In addition, the author cooperation network map showed that domestic cooperation in this field was frequent, but international cooperation was lacking. Therefore, it is urgent to strengthen international cooperation in the TIA field.

Journal analysis showed that the impact factors of the top 10 journals ranged from 1.889 to 5.284. We were considering that most journals were professional journals, with the characteristics of a small audience and narrow spread range. Therefore, the impact factor cannot be used as the sole criterion to judge the quality of research. Based on the JCR, we found 3 journals ranked in Q1 and 6 journals ranked in Q2. Therefore, we could still believe that most TIA-related articles had good research quality and were worth learning by researchers.

At the end of this study, we explored the hot spots and trends of TIA research. As we all know, the strength of a paper's citation burst represents the degree of concern by peers. This study found that the top 10 references with the strongest strength citation intensity focused on exploring the safety and efficacy of TXA in joint arthroplasty and the best strategies for its use. This result is consistent with the result of the co-citation analysis. Hence, the effectiveness and safety of TIA and the best strategies for using TXA are the current research hotspot and trend in the TIA field. Based on the previous researches related to TIA, many studies have demonstrated that intravenous (IV) administration of TXA and intraarticular (IA) administration of TXA can significantly reduce blood and transfusions without increase the risk of thrombosis in joint arthroplasty. During the recent years, many researchers have focused on the combination of IV and IA in joint arthroplasty, and some results demonstrated that the combination of IV and IA in joint arthroplasty had the positive effect on hemostasis and might be a preferable option (47, 48). However, some studies also had the opposite results (49). So for, there is still controversy about IV administration of TXA and IA administration of TXA in joint arthroplasty. Therefore, further researches are needed to explore the effect of IV administration of TXA and IA administration of TXA in joint arthroplasty.

The heat of research on effectiveness and safety of TIA has been enduring. In 2021, Zak et al. showed that topical and IV TXA were equally safe when used in patients undergoing total joint arthroplasty (TJA) procedures with a history of coronary artery disease (CAD) and coronary stenting (50). In 2021, a study by Poeran et al. classified patients undergoing total knee arthroplasty (TKA) surgery into high- and low-risk groups based on three defined conditions and explored the safety of TXA among patients in different risk groups. The results showed no significant increase in the incidence of various thromboembolic and ischemic complications among high-risk patients using TXA, and there was also a reduction in length of stay and hospital costs. Meanwhile, patients in the high-risk group who used high-dose TXA also showed a decreasing trend in the incidence of complications (51). In addition, a national database analysis in 2021 also suggested that TXA might play an essential role in reducing periprosthetic joint infection (PJI) after TJA (52). Therefore, the number of publications on the effectiveness and safety of TIA has remained high in the last 2 years, indicating this topic will continue to have significant research value and deserve the attention of researchers.

We also note that research on the optimal use strategy for TXA has gradually increased in recent years. Studies have demonstrated the high safety of both IA and IV (27, 30, 31), yet the efficacy of these two modes of administration is controversial. A study by Peng et al. in 2021 showed that in TKA, the IA administration of TXA significantly reduced total and occult blood loss without reducing drainage compared to IV (53). In 2021, Zhang et al. in a prospective study showed that intraoperative spraying and drug-soaked gauze to cover the wound combined with local injection into the articular cavity significantly reduced intraoperative blood loss, postoperative drainage, postoperative blood loss, total blood loss, and the incidence of deep vein thrombosis in patients undergoing TKA than intravenous drip combined with local injection into the articular cavity (54). This seemed to predict that IA administration of TXA will be the best choice in the TIA. However, studies have also shown that there is no statistically significant difference between IA and IV administration of TXA in reducing blood loss (35), and that IV administration is even more effective in preventing hemoglobin decline (55). The current controversy on this topic is clinically important and is attracting the attention of an increasing number of researchers. In summary, the effectiveness and safety of TIA and the best strategies for using TXA are the current research hotspot and trend in the TIA field.

This study also had many limitations. First, the data of the current study were extracted *via* software tools, which had the potential bias. Second, only data from WoSCC were included in this study, although WoSCC is the most widely and commonly used database for scientometric studies. Last but not least, the articles in 2022 were not included in our study, because the data were incomplete at the time of our database search.

## Conclusions

With the progress of population aging, the TIA field is receiving more and more attention. Based on bibliometric analysis and information visualization, the overall research trends and hot spots in this field can be determined, and necessary information can be provided for new researchers. Our research identified countries, institutions, authors, and journals that made significant contributions to this field over the last 20 years, as well as its limitations and deficiencies. Furthermore, this study confirmed that the efficacy and safety of TIA and the optimal strategy for using TXA are current or future research trends and hot spots in this field.

## Data availability statement

The original contributions presented in the study are included in the article/supplementary material, further inquiries can be directed to the corresponding author.

## Author contributions

JZ and RZ contributed to the conception and design of the project. JZ, RZ, YH, CX, HL, and HJ prepared the original draft. XL reviewed and edited the manuscript and supervised the study. All authors contributed to the article and approved the submitted version.

## Funding

The reported work was supported by the National Natural Science Foundation of China (NSFC) (Grant No. 8187090823) and Innovative Project for doctoral students of the First Affiliated Hospital of Chongqing Medical University (CYYY-BSYJSCXXM-202215).

## Conflict of interest

The authors declare that the research was conducted in the absence of any commercial or financial relationships that could be construed as a potential conflict of interest.

## Publisher's note

All claims expressed in this article are solely those of the authors and do not necessarily represent those of their affiliated organizations, or those of the publisher, the editors and the reviewers. Any product that may be evaluated in this article, or claim that may be made by its manufacturer, is not guaranteed or endorsed by the publisher.
